# A sequential toggle cell-SELEX DNA aptamer for targeting *Staphylococcus aureus*, *Streptococcus agalactiae*, and *Escherichia coli* bacteria

**DOI:** 10.1186/s43141-022-00374-9

**Published:** 2022-07-01

**Authors:** Arizah Kusumawati, Apon Zaenal Mustopa, I. Wayan Teguh Wibawan, Agus Setiyono, Mirnawati Bachrum Sudarwanto

**Affiliations:** 1 Study Program of Veterinary Public Health, IPB Graduate School, IPB University, Bogor, Indonesia; 2 Research Center for Biotechnology, National Research and Innovation Agency (BRIN), Cibinong, Bogor, 16911 Indonesia; 3 Department of Animal Diseases and Veterinary Public Health, Faculty of Veterinary Medicine, IPB University, Bogor, Indonesia; 4 Division of Medical Microbiology, Department of Animal Infectious Diseases and Veterinary Public Health, Faculty of Veterinary Medicine, IPB University, Agatis Street, Dramaga, Bogor, West Java 16680 Indonesia; 5 Department of Veterinary Clinic Reproduction and Pathology, Faculty of Veterinary Medicine, IPB University, Bogor, Indonesia

**Keywords:** DNA aptamer, STC SELEX, *E. coli*, *S. aureus*, *S. agalactiae*

## Abstract

**Background:**

Mastitis is an inflammation of the mammary glands caused by a microbial infection. The common bacteria causing this infection in dairy farms are *Staphylococcus aureus*, *Streptococcus agalactiae*, and *Escherichia coli*. The aptamer is a new biosensor platform for detecting pathogens; however, its use for simultaneous detection of *S. aureus*, *S. agalactiae*, and *E. coli* bacteria has not been reported. This study’s objective is to isolate and characterize polyclonal DNA aptamer with broad reactivity to the mastitis bacteria *S. aureus*, *S. agalactiae*, and *E. coli* using a sequential toggle cell-SELEX.

**Methods and results:**

The DNA aptamer pool from SELEX 15 was inserted into the pGEM-T easy plasmid. Furthermore, the transformant clones were selected by PCR colony, plasmid isolation, and sequencing. Six DNA aptamers, consisting of S15K3, S15K4, S15K6, S15K13, S15K15, and S15K20 with a constant region and the right size of 81 bp were derived from the sequencing analysis. The secondary structure of the DNA was predicted using Mfold software. The DNA was analyzed with binding characteristics, including binding capacity and affinity (Kd), using qPCR. The results indicated aptamer S15K15 has the highest binding ability into *S. agalactiae*, while S15K13 performed binding capacity most to *E. coli* EPEC 4, and S15K3 has the highest capacity of binding to *S. aureus* BPA-12.

**Conclusion:**

Aptamer S15K3 has the best binding characteristics on all three bacterial targets.

## Background

Mastitis is an inflammation of the mammary glands caused by a microbial infection. This disease causes large economic losses to farmers, adverse effects on livestock health, decreased quality and production of milk, the early slaughter of cattle, replacement costs for cattle, low selling prices, and additional costs for treatment livestock [1, 2]. Clinical mastitis is easily detectable due to symptoms, unlike subclinical mastitis, which is difficult to identify since the udder and milk do not exhibit clinical signs [1, 3]. Various gram-positive and gram-negative bacteria commonly cause mastitis are divided into contagious pathogens, *Staphylococcus aureus*, *Streptococcus agalactiae*, and *Mycoplasma* spp., and environmental pathogens, including *Escherichia coli*, *Enterococcus* spp., coagulase-negative *Staphylococcus*, and *Streptococcus uberis* [3–5]. The bacteria of *S. aureus*, *S. agalactiae*, and *E. coli* were then selected in this study because they cause mastitis and are often found in dairy farms [6–9].

Detection and identification of pathogens causing mastitis are carried out to establish the diagnosis of this infection and determine the route of spread, evaluate mastitis transmission, and assess prognosis and the number of affected cattle. Molecular diagnostic methods for mastitis pathogens identifications are based on PCR methods. The multiplex PCR technique has been used for the detection of multi-pathogenic mastitis simultaneously. Real-time PCR analysis identifies and quantifies mastitis pathogens in milk [10, 11]. The PCR method requires sample processing, relatively expensive equipment and materials, optimization reaction of PCR, and trained technicians [11, 12]. Therefore, developing sensitive, specific, effective, and efficient biomarkers is challenging for pathogen detection and diagnosis of mastitis. Biosensors use biological receptor molecules such as antibodies, enzymes, and nucleic acids that combine with transducers to generate signals indicating certain biological events, including antigen-antibody interactions [13, 14].

Aptamers are single-stranded DNA or RNA sequence fragments, where in vitro ligand selection is by systematic evolution of ligands by exponential enrichment (SELEX). Aptamers are short oligonucleotides made synthetically and easy to store. It substitutes antibodies that can recognize a wide range of diverse targets with several advantages, including more stability, simple synthesis, low cost, easy labeling, wide application, resistance to denaturation, and high sensitivity [15, 16]. The aptamer targets can be immunogenic or non-immunogenic, and they can be applied in biosensors, bioimaging, diagnostics, drug delivery, and therapeutics. The limitations of aptamers include low chemical diversity and susceptibility to degradation by nuclease. The drawbacks of aptamer can be overcome by various chemical modifications to increase the aptamer stability [17, 18]. Various studies using aptamers for the detection of *Staphylococcus aureus* [19–21], *Streptococcus* sp. [22], and *Escherichia coli* [23, 24] have been successfully conducted. However, the use for the simultaneous detection of *S. aureus*, *S. agalactiae*, and *E. coli* bacteria has not been reported. Therefore, this study was conducted to isolate and characterize polyclonal DNA aptamer with broad reactivity to *S. aureus*, *S. agalactiae*, and *E. coli* bacteria using a sequential toggle cell-SELEX (STC SELEX) method. Furthermore, the polyclonal DNA aptamer will be developed to simultaneously detect all three targets of mastitis pathogenic bacteria for easy and fast detection.

## Methods

### Bacterial strains and culture

The pathogenic bacteria of mastitis used in the SELEX process are *S. aureus*, *S. agalactiae*, and *E. coli* bacteria. Bacteria *S. aureus* BPA-12 and *E. coli* EPEC 4 were isolated from cow’s milk with subclinical mastitis [7], while bacteria *S. agalactiae* was obtained from the Faculty of Veterinary Medicine, IPB University. In addition, the *E. coli* DH5α (Invitrogen) bacteria were used for cloning. Single clones of bacteria were grown in the NB medium (*S. aureus*, *E. coli*) and BHI medium (*S. agalactiae*). The culture was incubated overnight at 37 °C with shaking 220 rpm, and the target cells bacteria for selection were subcultured at 37 °C to an OD_600_ of 0.3 (equivalent to 10^8^ bacteria/mL), washed twice with 1× PBS, and diluted into selection buffer of PBS containing 1.4 mM MgCl_2_.

### Random DNA library and primer

The single-stranded DNA (ssDNA) library incubated with the target bacteria has about 10^12^ random nucleotide sequences. The ssDNA library consisted of 81 base nucleotides with a central random region of 40 bases flanked by two constant regions, 21 bases at the 5′ regions and 20 bases at the 3′ regions. The DNA library sequence 5′-CCGGAATTCCTAATACGACTC-(N40)-TATTGAAAACGCGGCCGCGG3′ was synthetically made. A pair of primers for PCR amplification were forward 5′-CCGGAATTCCTAATACGACTC-3′ and reverse 5′-CCGCGGCCGCGTTTTCAATA-3′). Meanwhile, the primer T7 promoter 5′-TAATACGACTCACTATAGGG-3′ was used for sequencing.

### Sequential toggle cell-SELEX (STC-SELEX)

Random library nucleotides (ssDNA) were heated for 5 min at a temperature of 95 °C, cooled for 15 min at 25 °C, and incubated with 100 μL (10^7^) *E. coli* cells suspended in a selection buffer. The mixture was incubated under agitation 220 rpm at 30 °C temperature for 1 h. The bacterial suspension was centrifuged for 6 min at 8000 rpm. The unbonded ssDNA with *E. coli* in the supernatant were discarded by washing in 200 μL of 1× PBS twice. The ssDNA bound to *E. coli* was recovered by centrifugation at 8000 rpm for 6 min. Furthermore, the solution was eluted with 100 μL of sterile water, heated at 95 °C for 5 min to separate the bound ssDNA from bacterial cells. The ssDNA in the supernatant were recovered by centrifugation at 8000 rpm at 4 °C for 6 min. Then, the supernatant (eluate) was collected, and the concentration was measured using a NanoPhotometer. The eluate was amplified by the PCR method and was used in the next stage of the SELEX process.

The eluate was amplified using symmetric PCR to obtain dsDNA amplicon, followed by asymmetric PCR to get ssDNA amplicon. First, amplification of the eluate was conducted by symmetric PCR in a 40 μL reaction using NZYTaq Master Mix (NZYTech). The PCR conditions were 95 °C for 5 min with 20 cycles consisting of 95 °C for 45 s, 64 °C for 45 s, and 72 °C for 45 s; and 72 °C for 8 min. Afterward, agarose gel electrophoresis on 3% (w/v) was used to analyze PCR products. Finally, the PCR results were purified by Wizard® SV gel and PCR cleanup system (Promega) in a 25 μL elution. The condition of the asymmetric PCR process is the same as symmetrical PCR except for the ratio of forward:reverse primer (20:1) and the number of PCR cycles (30 cycles). Furthermore, asymmetric PCR results were analyzed using the same method as symmetric PCR [25].

The ssDNA aptamer isolated from the first round with *E. coli* was mixed with 10^7^*S. aureus* cells as the second target for the second selection round. The procedure for isolating the ssDNA bound to *S. aureus* was the same as the first round. Subsequently, the ssDNA aptamer was isolated against *S. agalactiae* following the same procedure sequentially. After one loop of isolation from *E. coli* to *S. agalactiae*, the ssDNA aptamer was incubated with *E. coli* again. This procedure was repeated for five loops of 15 isolation procedures.

### Cloning, selection, plasmid isolation, and sequencing

The cloning method consisted of several steps: preparation of insert, competent *E. coli* DH5α cells (Invitrogen), ligation, transformation, screening of transformant clones, isolation of recombinant plasmids, and sequencing of specific aptamers. The isolated ssDNA from the last round were amplified to have dsDNA to prepare insert for cloning. The PCR mix was ddH_2_O 19.2 μL, MyFi DNA Polymerase (Bioline) 1.6 μL, MyFi buffer (Bioline) 8 μL, primers forward 1.6 μL, primers reverse 1.6 μL, and DNA template 8 μL with a total volume of 40 μL. Additionally, the PCR conditions were 95 °C 1 min; 25 cycles consisting of 95 °C 15 s, 64 °C 15 s, and 72 °C 15 s; and 72 °C 8 min. Agarose gel electrophoresis on 3 % (w/v) was used to analyze PCR products. It was stained by ethidium bromide staining and visualized under a UV transilluminator. The target dsDNA (81 bp) band was cut and purified using Wizard® SV gel and PCR cleanup system (Promega).

The insert (dsDNA aptamer 81 bp) was cloned using a pGEM-T easy Cloning Kit (Promega), and the ligation mix was 5 μL 2× rapid ligation buffer, 1 μL plasmid pGEM-T easy (Promega), 1 μL T4 DNA ligase, and 3 μL dsDNA insert (DNA aptamer) with a total volume of 10 μL. The ligation was incubated overnight at 4 °C, and the result was transformed into *E. coli* DH5α competent cells using heat shock method at 42 °C for 90 s. The transformation results were then cultured on a selection medium (LB/Amp/X-Gal/IPTG) and incubated at 37 °C for 18 h. The transformant clones were selected by PCR colony, plasmid isolation, and sequencing. White colonies were chosen randomly, while the plasmid DNA was purified using a High-Speed Plasmid Mini Kit (Geneaid). Finally, the inserted fragments were confirmed using PCR amplification, and the sequencing of the aptamer candidates was conducted at 1st base to identify specific aptamer sequences.

### Secondary structure prediction and binding characteristics assays

The secondary structure of DNA aptamer was predicted through in silico analysis using the Mfold software program [26], and the set conditions used in binding assays include 1.4 mM MgCl_2_ and 137 mM NaCl, at 30 °C. The G-quadruplex secondary structures prediction was performed by the QGRS Mapper software program [27, 28].

For the binding capacity assay, washed bacterial cells (10^8^) were incubated with 200 nM aptamers for 45 min at 30 °C under constant agitation 220 rpm vortex every 15 min. The mixture was centrifuged at 8000 rpm for 6 min, and the supernatant was discarded. The bacterial cells were then washed with 500 μL selection buffer by centrifugation at 8000 rpm for 6 min. The bacterial cells were resuspended in 100 μL of ddH_2_O and heated at 95 °C for 5 min to elute bound aptamers. Finally, the mixture was centrifuged at 13,000 rpm for 10 min 20 °C to harvest bound aptamers in the supernatant, and the quantification cycle (Cq) value bound to the cells was carried out by qPCR (MyGo Pro real-time PCR) analysis using SYBR Green. Ten microliters qPCR reaction was set up containing 1 μL template (bound aptamers), 5 μL of THUNDERBIRD™ SYBR® qPCR Mix (Toyobo), 0.12 μL forward primer, 0.12 μL reverse primer, and 3.76 μL of ddH_2_O. The qPCR conditions for this process were 95 °C for 5 min; 20 cycles consisting of 94 °C for 45 s, 64 °C for 45 s, and 72 °C for 45 s; and 72 °C for 8 min.

For the binding affinity assay, bacteria cells (≈10^8^ bacterial cells/ml) were incubated with aptamers prepared in concentrations 100 nM, 200 nM, and 400 nM. The quantification of relative fluorescence units (RFU) was conducted by qPCR analysis using SYBR Green. The thermal cycle conditions were the same as binding capacity analysis. Furthermore, the Kd values for each aptamer were estimated by qPCR in nonlinear regression Lineweaver-Burk analysis [23], using the formula 1/[complex] = Kd/[Cmax] × 1/[aptamer] + 1/[Cmax], where Kd is the steady-state dissociation constant, [Complex] is the concentration of the bacteria-aptamer complex, and [Cmax] is the concentration of the complex at maximal binding capacity. The aptamer occupies all the binding sites, and [aptamer] represents the concentration.

## Results

### Sequential toggle cell-SELEX (STC-SELEX)

**Fig. 1:**
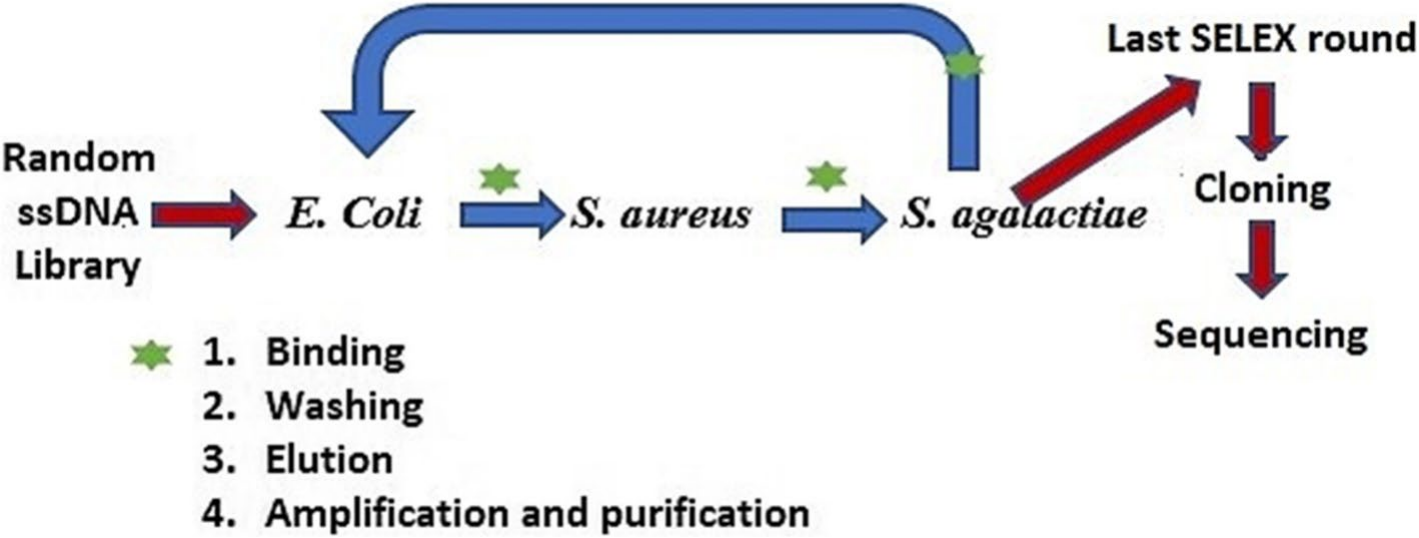
STC-SELEX process

The STC-SELEX process was initiated for aptamer isolation using a DNA library consisting of random fragments of ssDNA. *E. coli* cells were incubated with the DNA library in the first round of in vitro selection, and an isolation step was performed to obtain DNA bound to the target. Unbound DNA is removed by washing, and DNA bound to the target is eluted. The amplification of eluate was then carried out using symmetric and asymmetric PCR to obtain ssDNA for the next selection round. The enriched ssDNA bound to *E. coli* were incubated with *S. aureus* in the second-round selection. Meanwhile, those bound to *S. aureus* were incubated with *S. agalactiae* in the third selection round. This cycle was repeated using targets *from E. coli* to *S. aureus* and *S. agalactiae* five times (5 toggle loops) until the desired ssDNA aptamer pool was obtained. The SELEX process is carried out for 15 rounds to get an aptamer with a high affinity for all targets. The screening procedure for single-stranded DNA (ssDNA) aptamers with a broad affinity to three bacteria is illustrated in Fig. 1.

### Cloning, selection, plasmid isolation, and sequencing

**Fig. 2:**
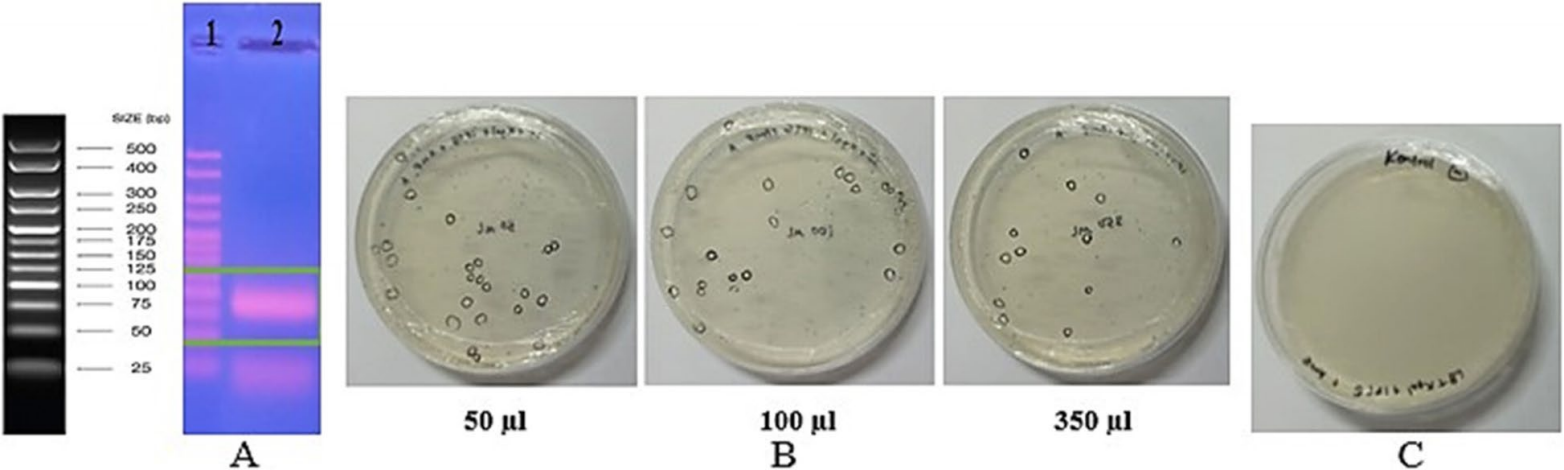
Preparation of insert and the results of transformation to *E. coli* DH5α. **A** 1 Marker. 2 Insert (DNA aptamer pool SELEX 15); **B** Transformed colony. **C** Negative control

**Fig. 3:**
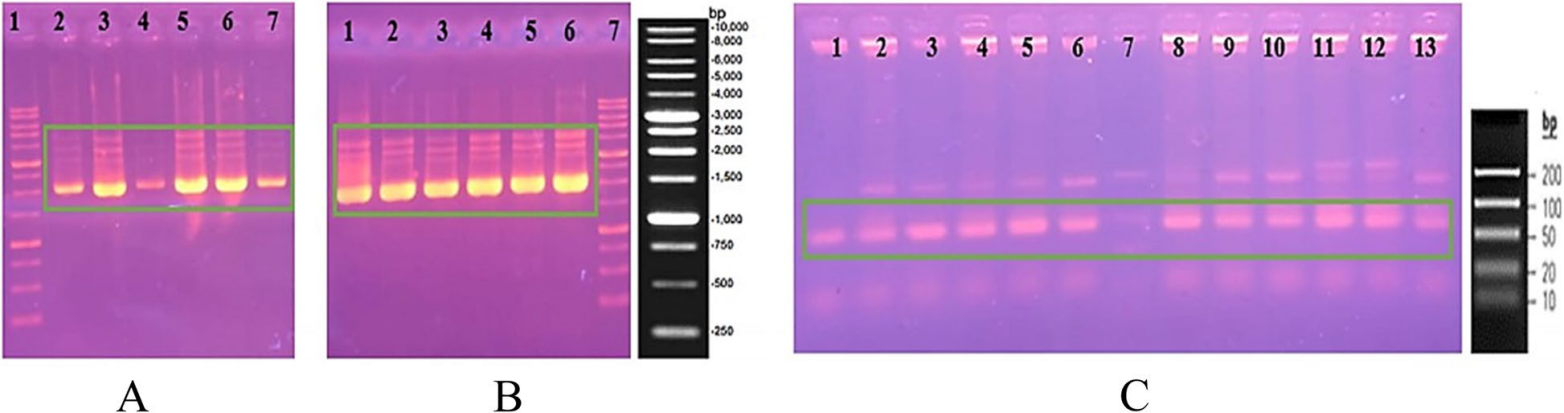
Electrophoretic of isolation plasmid and amplification PCR. **A** Electrophoretic of isolation plasmid. 1 Marker 1 kb, 2 S15K1, 3 S15K3, 4 S15K5, 5 S15K13, 6 S15K15, 7 S15K17. **B** Electrophoretic of isolation plasmid. 1 S15K2, 2 S15K4, 3 S15K6, 4 S15K12, 5 S15K16, 6 S15K20, 7 marker 1 kb. **C** Electrophoretic of amplification PCR. 1 S15K1, 2 S15K3, 3 S15K5, 4 S15K13, 5 S15K15, 6 S15K17, 7 marker low range, 8 S15K2, 9 S15K4, 10 S15K6, 11 S15K12, 12 S15K16, 13 S15K20

PCR amplified the last SELEX round of selection (SELEX 15) products, and the results show a band of 81 bp was obtained and cloned to *E. coli* DH5α. The transformation results were obtained in many transformant colonies white and blue on the selection medium (Fig. 2). Twenty white transformant clones were randomly selected for the PCR colony. Twelve selected white transformant clones were isolated by recombinant plasmid and amplified by PCR. The amplification results were obtained for all recombinant plasmid containing insert on 81-bp size (Fig. 3). The thick bands obtained from 9 clones were sequenced to determine the nucleotide bases. Subsequently, the nine sequences of DNA aptamer were aligned using BioEdit software and obtained 6 DNA aptamers with the correct constant region and size of 81 bp. Table 1 summarizes the six ssDNA sequences after STC-SELEX with different 40 bases of the central random region. Table 2 shows a G-quadruplex forming motif of the 6 DNA aptamer, and S15K15 does not have a G-quadruplex forming motif.

**Table 1:** ssDNA aptamer sequences isolated using the STC-SELEX process

ID	Sequence (5ˊ-3ˊ)	Size (bp)
**S15K3**	CCGGAATTCCTAATACGACTCCCAGCAGCAAGGTGCGGTACCCGGGGATGCGGGCTTGCTGTATTGAAAACGCGGCCGCGG	81
**S15K4**	CCGGAATTCCTAATACGACTCCCCGGGCCCACAGGGTACGCGTCTGCGGCTGGCCGGTCCCTATTGAAAACGCGGCCGCGG	81
**S15K6**	CCGGAATTCCTAATACGACTCGCGGGACGGGGAGTGCGCTGGGCATGTGGGCGCCGGGGGATATTGAAAACGCGGCCGCGG	81
**S15K13**	CCGGAATTCCTAATACGACTCCACGCGCAGGCAGCCACCGACCAGGTGCTCGTATGGTTGGTATTGAAAACGCGGCCGCGG	81
**S15K15**	CCGGAATTCCTAATACGACTCCAGGACAGTACTCTGGACGGCAATACGTATATACGTACGGTATTGAAAACGCGGCCGCGG	81
**S15K20**	CCGGAATTCCTAATACGACTCCCGGCGCCACGACATGGGCGCTGCCGGTGTGGTCGCGGGATATTGAAAACGCGGCCGCGG	81

**Table 2:** QGRS sequences

Aptamer	QGRS sequences overlaps not included	length	G-score
**S15K3**	GGTGCGGTACCCGGGGATGCGG	22	18
**S15K4**	GGGCCCACAGGGTACGCGTCTGCGGCTGG	29	12
**S15K6**	GGGAGTGCGCTGGGCATGTGGGCGCCGGGG	30	39
**S15K13**	GGTTGGTATTGAAAACGCGGCCGCGG	26	11
**S15K15**	-	-	-
**S15K20**	GGCGCTGCCGGTGTGGTCGCGG	221	17

**Fig. 4:**
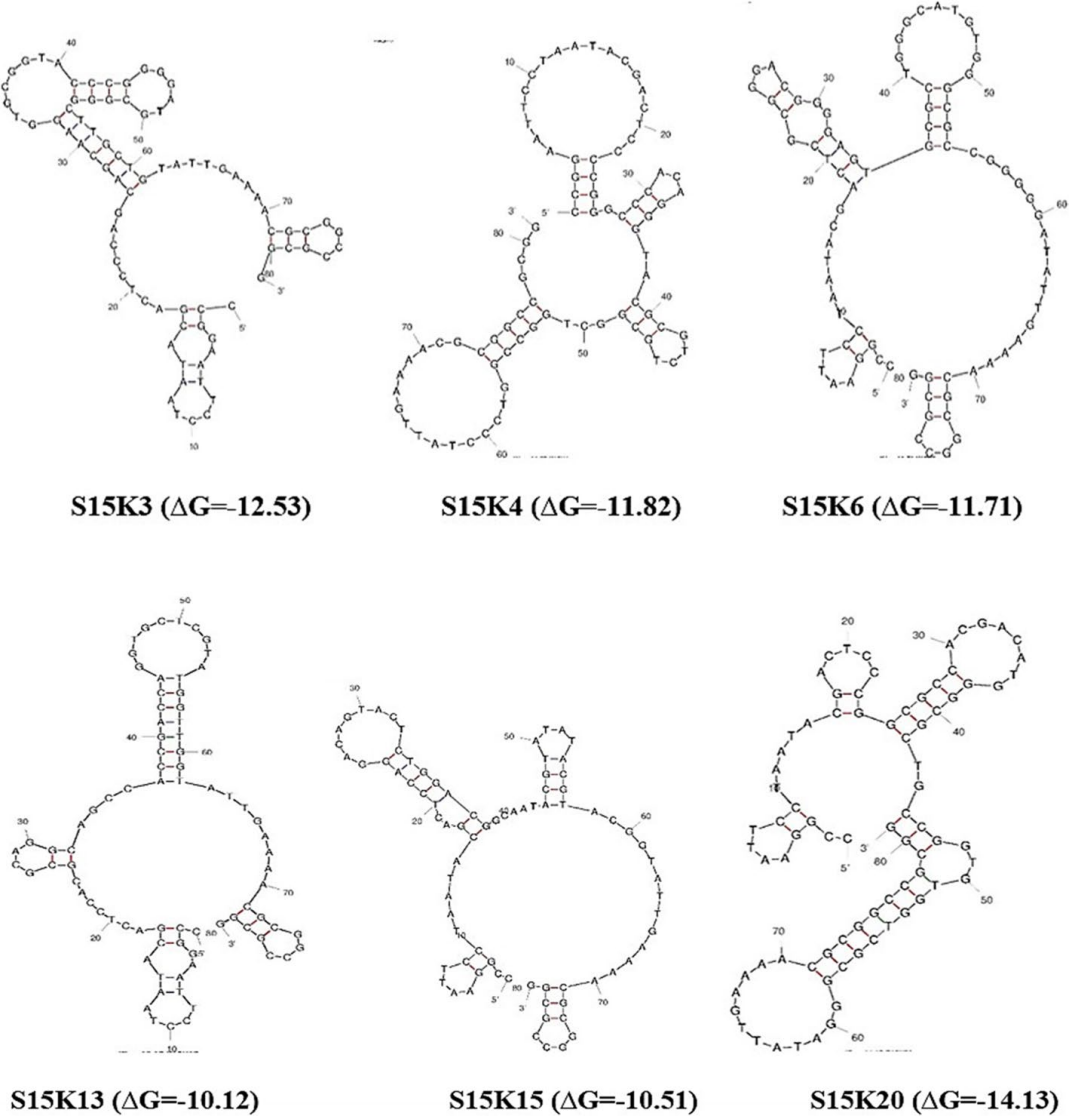
Prediction of DNA aptamer secondary structures

The six aptamers of S15K3, S15K4, S15K6, S15K13, S15K15, and S15K20 showed variables on loops structures (Fig. 4). The structures of S15K3 have 1 external, 3 hairpin, 1 interior, and 1 bulge loop, while the structures of S15K4 have 1 external and 4 hairpin loops. The S15K6 and S15K13 have 1 external, 4 hairpin, and 1 interior loop, while S15K15 and S15K20 have 1 external, 4 hairpins, and 1 bulge loop.

### Secondary structure prediction and binding characteristics assays

**Fig. 5:**
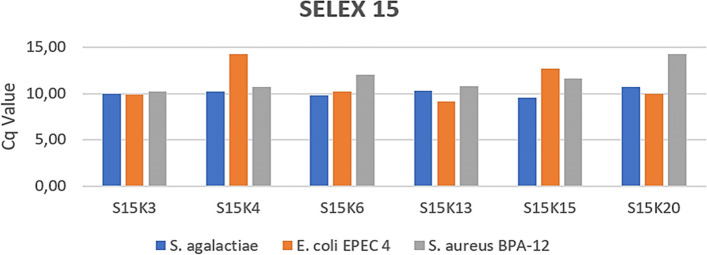
The binding capacity of the selected aptamers

The binding capacity of S15K3, S15K4, S15K6, S15K13, S15K15, and S15K20 with *S. agalactiae*, *E. coli* EPEC 4, and *S. aureus* BPA-12 was measured (Fig. 5). Figure 5 shows that aptamer S15K15 has the highest binding capacity to *S. agalactiae*, aptamer S15K13 to *E. coli* EPEC 4, and aptamer S15K3 to *S. aureus* BPA-12. The results showed that aptamer S15K3 has the best binding capacity on all three bacterial targets. The binding affinity (Kd) of S15K3, S15K6, S15K13, and S15K15 with *S. agalactiae*, *E. coli* EPEC 4, and *S. aureus* BPA-12 was measured (Table 3). Table 3 showed that S15K3 and S15K13 have a high binding affinity for *S. agalactiae*. The aptamer S15K6 has the highest binding affinity to *S. aureus* BPA-12, while S15K13 and S15K15 have high binding affinity to *E. coli* EPEC 4.

**Table 3:** The binding affinity of the selected aptamers

Aptamer	***S. agalactiae***	***S. aureus*** BPA-12	***E. coli*** EPEC 4
**Kd**			
**S15K3**	6,84 nM	36,70 nM	28,06 nM
**S15K6**	20,79 nM	17,01 nM	31,99 nM
**S15K13**	6,53 nM	60,69 nM	5,21 nM
**S15K15**	45,36 nM	20,90 nM	8,89 nM

## Discussions

The STC-SELEX method uses more than one target, and the selection process is carried out on different targets alternately in each SELEX round. It is applied to unlimited targets, including closely or distantly related bacterial strains, through in vitro selection. STC-SELEX technique is applied to target groups to identify widely reactive aptamers [29]. The polyclonal selection strategy produces aptamers capable of binding to multiple targets. This is achieved by incubating previously selected aptamers with the second and subsequent targets until other regions for binding are identified [30]. Toggle cell-SELEX selects aptamers with cross-reaction among targets, in contrast to conventional cell-SELEX, which isolate aptamers to a single target. Furthermore, aptamers generated from toggle cell-SELEX bind to common receptors or homologous receptors expressed among different target cells in the selection process. At the end of the selection, the obtained aptamer recognizes the target cells used in the selection process [31].

The secondary structure of DNA aptamer was predicted by utilizing Mfold software to analyze the aptamer sequences’ impact while binding. Negative Gibbs free energy (∆G) indicates that the formation of the secondary structure of each DNA aptamer occurs spontaneously. Aptamers fold into unique secondary structures with Watson-Crick as basic in the Mfold software. The secondary structure selected was the most thermodynamically stable, as indicated by the minimum fold free energy [28, 32]. Meanwhile, differences in the composition of nucleotide bases affect the formation of various secondary aptamer structures such as stem, loop, bugle, pseudoknot, triplex, G-quadruplex, and hairpin [26, 33]. The stem-loop structures of aptamers are important as binding motifs in the target recognition [34]. They have a unique conformation capable of specific binding to targets through interactions or combinations of van der Waals bond formation, hydrogen bonds, electrostatic interactions, and complementary forms with a binding affinity (Kd) ranging from picomolar to nanomolar [35, 36].

The last in silico test uses quadruplex forming G-rich sequences (QGRS) Mapper software program to analyze putative motifs in nucleotide sequences [37]. G-quadruplex (G4) is a 3D structure consisting of tetrads of Gs (guanine) linked by Hoogsteen-type hydrogen bonds. The number of tetrads in the quadruplex is determined by the size of the G-group [27, 38]. QGRS Mapper program uses a scoring system to predict quadruplex’s existence to form G-rich sequences in nucleotide. G-scores greater than zero represented the possibility of forming G-quadruplex, where a higher value represents better quadruplex-forming candidates [32]. The DNA aptamers have been reported to have strong and specific binding for various targets, especially proteins, because of their thermodynamically and chemically stable structure. Furthermore, the development of aptamer technology has shown the G-quadruplex structure’s potential as a promising molecular tool in targeting biologically important ligands and potentially becoming a diagnostic and therapeutic alternative to antibodies [39, 40].

The binding capacity and dissociation constant (binding affinity-Kd) are the two most important parameters to quantify the aptamers binding to a target [41]. Meanwhile, the entire binding process is sensitive to the ionic environment [42]. The measurement of binding characteristics is often conducted under distinct conditions, leading to analytical difficulties of their application for biosensors [43].

Selected polyclonal DNA aptamers will be applied for the simultaneous detection of *S. aureus*, *S. agalactiae*, and *E. coli* bacteria in diagnosing subclinical mastitis using dot blot method. Dot blot assay as a detection system is a fast, simple, and inexpensive, and the results are visible to the naked eye. The main step method of aptamer-based dot blot assay is similar to antibody-based dot blot assay [44]. The dot blot test is easy, rapid, and suitable for field applications because there is no need for highly trained technicians or expensive equipment [45]. Paper-based sensors are powerful tools for detecting pathogens because they are affordable, sensitive, specific, easy to use, fast, and robust [46].

## Conclusions

The STC-SELEX for targeting *S. aureus*, *S. agalactiae*, and *E. coli* in 15 rounds (5 toggle loops) obtained 6 DNA aptamers consisting of S15K3, S15K4, S15K6, S15K13, S15K15, and S15K20. The secondary structure and QGRS sequences were predicted using the software. Furthermore, the analysis of binding capacity and affinity (Kd) using qPCR showed that aptamer S15K3 has the best binding characteristics on all three bacterial targets.

## Data Availability

All data generated or analyzed during this activity are included in this published article.

## Abbreviations

STC-SELEX: Sequential toggle cell systematic evolution of ligands by exponential enrichment
PCR: Polymerase chain reaction
DNA: Deoxyribonucleic acid
RNA: Ribonucleic acid
EPEC: Enteropathogenic *Escherichia coli*
NB: Nutrient broth
BHI: Brain-heart infusion broth
PBS: Phosphate-buffered saline
Kd: Dissociation constant
QGRS: Quadruplex forming G-rich sequences
